# Evaluation of the Effects of Colostrum Substitutes on IgG Levels and Humoral Immune Development in Polypay Lambs

**DOI:** 10.3390/vetsci12111075

**Published:** 2025-11-10

**Authors:** Clay Schoen, Blake Johnson, Steven Lawson, Rosemarie Nold, Christopher Chase, Benoit St-Pierre, Manuel Alexander Vasquez-Hidalgo

**Affiliations:** 1 Department of Veterinary and Biomedical Sciences, South Dakota State University, Brookings, SD 57007, USA; clayschoen@outlook.com (C.S.); steven.lawson@sdstate.edu (S.L.); christopher.chase@sdstate.edu (C.C.); 2 Department of Animal Science, South Dakota State University, Brookings, SD 57007, USA; blake.johnson@sdstate.edu (B.J.); rosemarie.nold@sdstate.edu (R.N.); benoit.st-pierre@sdstate.edu (B.S.-P.)

**Keywords:** colostrum, IgG, lambs, ovalbumin

## Abstract

**Simple Summary:**

The objective of this study was to investigate how different colostrum sources impact immunity and immune development in Polypay lambs. Newborn lambs were assigned to four groups: fresh ewe colostrum (FrC; *n* = 10), frozen ewe colostrum (FZ; *n* = 11), frozen cattle colostrum (CC; *n* = 11), or artificial cattle colostrum (AC; *n* = 11). Lambs received the colostrum immediately after birth and were raised on milk replacer, creep feed, and hay. Immunoglobulin G (IgG) was measured at birth and then weekly until 28 days of age (d). Lambs were immunized with 1 mL ovalbumin (chicken egg protein used to stimulate the lambs’ immune system) at 35 and 63 d. Ovalbumin-specific antibodies were quantified. Fresh ewe colostrum yielded higher relative IgG at later time points (7, 14, 21, and 28 d) compared to FZ. Frozen cattle colostrum resulted in higher IgG concentrations than AC at 24 h, 7, 14, and 21 d. Fresh ewe colostrum and AC showed a faster and more robust (more than double) ovalbumin antibody response compared to CC lambs 2 to 8 weeks post immunization. Our results suggest that the best colostrum alternative for newborn lambs is frozen ewe colostrum.

**Abstract:**

This study investigated colostrum source impact on passive immunity transfer and humoral immune development in Polypay lambs. Newborn lambs (4.80 ± 0.33 kg BW) were assigned to four groups: fresh ewe colostrum (FrC; *n* = 10), frozen ewe colostrum (FZ; *n* = 11), frozen cattle colostrum (CC; *n* = 11), or artificial cattle colostrum (AC; *n* = 11). Lambs received 65 mL/kg colostrum within 4 h post-parturition and were raised on milk replacer, creep feed, and hay until weaned (28 days of age [d]). Immunoglobulin G concentrations were measured at birth and then weekly until 28 d. Lambs were immunized with 1 mL ovalbumin (2 mg/mL PBS) at 35 and 63 d. Ovalbumin-specific antibodies were quantified. A tendency (*p* = 0.06) suggested FrC yielded higher relative IgG at later time points (7, 14, 21, and 28 d) compared to FZ. Frozen cattle colostrum resulted in significantly (*p* = 0.02) higher IgG concentrations than AC at 24 h, 7, 14, and 21 d. Fresh ewe colostrum and AC showed a faster (1 week-post-immunization) and more robust (>175%; *p* ≤ 0.07) ovalbumin humoral response compared to CC lambs 2 to 8 weeks-post-immunization. Fresh ewe colostrum seems to provide the best passive and adaptive immunity compared to other colostrum sources. Moreover, our results suggest that the best colostrum alternative for newborn lambs is frozen ewe colostrum.

## 1. Introduction

The United States sheep industry spans a broad geographical range with vast production systems from coast to coast. The industry has an estimated 1.4 billion dollars in total economic output [1]. Despite the variation across the country, most death losses in the industry occur within the first week of life. Furthermore, seventy-nine percent of those losses occur within the first four days following birth, with starvation and respiratory disease being the leading contributors [2]. Both can be reduced if lambs receive adequate amounts of colostrum [3].

Colostrum is the first milk produced by the dam following parturition. In sheep, colostrum provides lambs with essential nutritional components, including fats and proteins, as well as immunological components of immunoglobulins, cytokines, hormones, and maternal leukocytes [4,5,6]. The transfer of maternal immune components is known as passive transfer. Ewe colostrum contains 3 major isotypes of antibodies: IgG, IgA, and IgM [7]. The IgG concentration of ewe colostrum can vary by breed and parity [8,9]. Conversely, freezing ewe colostrum seems to not have an effect on the amount of IgG absorbed by the lamb [10]. In sheep, IgG makes up to 92% of the total colostral immunoglobulins with IgA representing 6% and IgM representing 2% [7]. Moreover, IgM and IgA are absorbed less efficiently; much of their protective role is local (gut mucosal protection) rather than systemic [7]. Therefore, IgG is the most impactful immunoglobulin for establishing passive immune function. Passive transfer of immunoglobulins, particularly immunoglobulin G (IgG), provides the lamb with immune protection during the early stages of life. In calves, passive transfer of cytokines and maternal leukocytes also promotes the development of the neonate’s immune system [11,12].

Given the importance of colostrum, when a ewe gives birth and does not produce enough colostrum, producers often substitute with other sources. In Spain one study that compared goat vs. sheep colostrum in newborn lambs found no differences in IgG concentration or complement system activity [13]. Traditionally, sheep producers in the United States replace a dam’s colostrum with fresh colostrum from another ewe, preferably one that has delivered a single lamb [9]. If available, frozen ewe colostrum—sourced either from the same farm or externally—is commonly used as an alternative [9]. In goats, frozen colostrum seems to provide the same IgG levels as refrigerated colostrum [14]. However, it is not clear if the same occurs in Polypay sheep. When neither fresh nor frozen ewe colostrum is accessible, producers resort to feeding newborn lambs fresh or powdered bovine colostrum to provide at least minimal immune protection [9]. Bovine colostrum has greater levels of IgGs compared to sheep colostrum [15]. However, despite the use of these colostrum substitutes within the United States industry, little scientific data is published on how they affect passive immune protection or their ability to promote the development of the lamb’s immune system.

Ovalbumin (OVA) is a large extracellular protein commonly found in chicken egg. Since ovalbumin is found only within avian egg whites, the immune system of most animals recognizes it as a foreign antigen. Therefore, ovalbumin is effective at stimulating a robust humoral immune response, making it useful for assessing the capacity of the host for specific antibody production [16]. In calves, ovalbumin stimulated humoral response has been successfully used to assess immune system development [17,18,19]. To our knowledge, this method of assessing humoral response has not been widely used in lambs.

This study was conducted to assess the effects of fresh ewe colostrum, frozen ewe colostrum, frozen cattle colostrum and artificial cattle colostrum on passive immune protection (IgG levels) and the development of the humoral immune system of Polypay lambs. The hypothesis was that lambs receiving fresh colostrum would have greater total and relative IgG concentrations, as well as show slower IgG decay than other colostrum sources. Furthermore, the hypothesis was that fresh ewe colostrum would promote a faster development of the neonatal humoral immune response, resulting in a faster and greater antibody response to ovalbumin.

## 2. Materials and Methods

### 2.1. Animal Handling and Sample Collection

All procedures involving the use of animals in this study were approved by the South Dakota State University (SDSU) Institutional Animal Care and Use Committee (Approval Number 2307-062A).

Forty-three Polypay lambs born as twins or triplets were used for this study. Immediately after birth, lambs were dried with towels and umbilical cords were trimmed and dipped with a 7% iodine solution. Lambs were weighed; blood was collected via jugular venipuncture and fecal samples were collected. Lambs were randomly assigned to one of four treatment groups: Fresh ewe colostrum (FrC), Frozen ewe colostrum (FZ), Frozen bovine colostrum (CC), and Artificial bovine colostrum (AC). There was no difference (*p* = 0.40) in birthweight between treatment groups. Fresh colostrum was sourced from the dam immediately before feeding the lamb (Table 1). Frozen ewe colostrum sourced from the South Dakota State University (SDSU) sheep unit and other flocks within the Midwest was collected for 1–3 months before the start of the experiment, then pooled and stored at −20 °C (150 mL aliquots; Table 1). Frozen bovine colostrum was sourced from a dairy farm (Mills Dairy Farm LLC, Hayesville, OH, USA), pooled and stored at −20 °C (150 mL aliquots). Powdered artificial colostrum containing freeze-dried bovine IgG (Shepherd’s Choice Premium Colostrum Replacer, Washington, IA, USA) was used for the AC group.

Once assigned to a treatment group, lambs were fed colostrum two times. Lambs were fed 65 mL of colostrum per kilogram of birth weight via an esophageal tube within the first 4 h of life; the maximum amount of colostrum given in one feeding did not exceed 177 mL. Lambs were then given a second feeding 90–120 min following the first feeding. Thereafter, all lambs were housed in a group pen where they had access to ad libitum warm milk replacer (The Shepherd’s Choice Lamb and Kid Instant Milk Replacer, Washington, IA, USA). Lamb weight, blood and fecal samples were collected 24 h post colostral ingestion and then at 7, 14, 21, and 28 days of age (D). Blood samples were allowed to rest for one hour, then were centrifuged (3010× *g* for 20 min), serum was collected and stored at −20 °C until further analyses. Fecal samples were frozen (−20 °C) and stored until further analyses. At 7 D, lambs were docked and male lambs castrated using an elastrator and a band. Additionally, starting at 7 D, lambs were offered access to ad libitum creep feed (18% Crude Protein, texturized) and warm milk replacer. At 14 D, lambs were transitioned to ad libitum cold milk (1–4 °C), creep feed, and alfalfa hay (16% Crude protein). These same conditions were kept until 28 D. At 28 D, lambs were abruptly weaned from milk replacer and offered ad libitum creep feed, and alfalfa hay. Ad libitum sodium bicarbonate was also offered at this time to reduce the risk of ruminal acidosis.

At five weeks of age, lambs (35 D) received bilateral subcutaneous cervical injections of 1 mL ovalbumin (Sigma-Aldrich, St. Louis, MO, USA; 2 mg/mL in PBS) homogenized with 1 mL of Freund’s Incomplete Adjuvant (ThermoFisher Scientific, Waltham, MA, USA). Blood samples were collected pre-immunization and weekly for 4 weeks post-immunization. Four weeks after the first immunization (63 D), lambs received a booster injection of 1 mL ovalbumin (2 mg/mL in PBS) homogenized with 1 mL of Freund’s Incomplete Adjuvant. Blood samples were collected weekly for 4 weeks following the second immunization. Weights were measured at weaning and 90 D.

### 2.2. IgG Detection

Serum and colostrum IgG concentrations were measured using Enzyme-Linked Immunosorbent Assay (ELISA) and Radial Immunodiffusion (RID). Serum and colostrum samples from all treatment groups were analyzed following the protocol described by the Ovine IgG ELISA kit (ALPCO, Salem, NH, USA). Ovine IgG concentrations of each sample were determined from optical densities using a set of known standards and a 5-parameter logistic curve. A cross-reactivity check was performed with the Ovine IgG ELISA to assess for cross-detection of bovine IgG. Samples from the treatment groups receiving cattle colostrum and artificial colostrum were also analyzed following the protocol described by the Bovine IgG ELISA kit (ZeptoMetrix, Buffalo, NY, USA). Bovine IgG concentrations of each sample were determined using a set of known standards and a 5-parameter logistic curve. A cross-reactivity check was performed on the Bovine IgG ELISA to assess for the detection of Ovine IgG. Radial immunodiffusion plates (JJJ Diagnostics, Bellingham, WA, USA) were used to quantify both ovine and bovine IgG levels. Colostrum and serum samples from all treatment groups were analyzed using the protocol described by the Ovine IgG RID plates kit (JJJ Diagnostics, Bellingham, WA, USA). Ovine IgG concentration of the unknown samples was determined using the diffusion radius of three known concentrations to create a linear regression equation. Colostrum and serum samples from the treatment groups receiving cattle and artificial colostrum were analyzed using the protocol described in the Bovine IgG RID plates kit (JJJ Diagnostics, Bellingham, WA, USA). Bovine IgG concentration of the unknown samples was determined using the diffusion radius of three known standard concentrations to create a linear regression equation.

### 2.3. Anti-Ovalbumin Antibody Detection

Anti-ovalbumin antibody level detection was performed using an indirect ELISA developed in the Veterinary and Biomedical Science department of SDSU using the methodology described in Ferrin et al., 2004, Okda et al., 2015, and Okda et al., 2016 [20,21,22]. Briefly, the ELISA was performed using an Immulon 1B 96-well plate (Fisher Scientific, Waltham, MA, USA). Antigen coating was performed by adding 100 uL of ovalbumin (OVA; 1 mg: 400 mL Antigen Coating Buffer [pH 9.6]), into the wells of odd-numbered columns. Even-numbered columns had 100 uL of Antigen Coating Buffer (pH 9.6) added, to serve as the negative control. Plates were then incubated for 1 h at 37 °C, followed by overnight incubation at 4 °C. Plates were then washed four times with 1× phosphate-buffered saline + 0.01% Tween 20 (PBST; Avantor, Radnor, PA, USA). After washing, 200 uL of 5% non-fat dry milk (Shurfine Nonfat Dry Milk, Shurfine Food, Burwell, NE, USA) was diluted in PBST, also referred to as blocking antibody diluent (BAD), and incubated for 1 h at 37 °C. Plates were then rewashed with PBST four times. Serum samples were diluted 1:100 in BAD, and 100 μL of each diluted sample was loaded in duplicate (1 and 1) into the odd-numbered column and the even-numbered control column. A known positive serum reference sample was created by pooling known positive serum samples. The known positive reference sample was diluted 1:100 in BAD and added to columns 1 and 2 of each plate. Plates were incubated for 1 h at 20 °C, then washed four times with PBST. Donkey anti-sheep horseradish peroxidase conjugated antibody (Jackson ImmunoResearch, West Grove, PA, USA) was diluted 1:20,000 in 1× PBS, then 100 uL was added to each well. Plates were incubated for 1 h at 20 °C, then washed four times with PBST. Next, 100 uL of 3,3′,5,5′-Tetramethylbenzidine (TMB; Avantor, Radnor, PA, USA) was added as the working substrate to each well. The reaction continued until one of the wells reached an optical density of approximately 2.0. The reaction was then stopped by adding 100 uL of 2N H_2_SO_4_ to each of the wells. Checkerboard titration experiments focused on determining optimal serum dilution in relation to the secondary HRP detection antibody in order to achieve the highest signal-to-noise ratio of signal. Optical densities (OD) were then measured using a plate reader set at 450 nm. Optical density values of the control cells were subtracted from the ODs of the coated cell (OD odd cell—OD of even cell) to normalize the data and control for background noise. Sample to Positive ratios (S/P) were calculated by dividing the OD of the sample by the OD of the known positive and multiplying by an arbitrary value of 3 as described in the following formula: S/P = [optical density (OD) of sample—OD of buffer/OD of positive control—OD of buffer] × 3. The multiplication by 3 is done because the pooled positive reference standard used for sample normalization demonstrated a high level of antibodies, therefore, the S/P ratio is multiplied by an arbitrary three-fold value for graphical purposes. The Specificity of the assay (performed by false positive testing) was determined as a part of our preliminary validation and quality control whereby over 100 known negative serum samples were initially run on the ELISA to establish a baseline negative and relative cutoff point. In addition, a positive control quality standard was produced by taking a serum pool from animals that were seropositive at 14 days post-vaccination to establish a standard in which a mathematical S/P ratio could be generated. This particular pool served as the reference standard that served as the positive control function of the equation.

### 2.4. Microbial DNA Extraction and PCR Amplification of the 16S rRNA Gene

A bead beating plus column method, which included the QIAamp DNA Mini Kit (Qiagen, Hilden, Germany), was used to extract microbial genomic DNA from individual fecal samples (*n* = 18) as previously described [23]. The universal forward 27F-5′AGAGTTTGATCMTGCTCAG [24] and reverse 519R-5′GWATTACCGCGCGCGCTG [25] primers were used to target the V1—V3 regions of the bacterial 16S rRNA gene by PCR to generate amplicons for DNA sequencing. Polymerase chain reaction and Next Generation Sequencing (Illumina MiSeq 2 × 300 platform, San Diego, CA, USA) services were performed by Molecular Research Laboratory (‘MRDNA’, Shallowater, TX, USA).

### 2.5. Bacterial Composition Analyses

Sequence data were analyzed using a combination of custom-written Perl scripts and publicly available software as previously described [26]; for a more detailed description of the procedure, please consult the Supplementary Materials [27,28,29,30,31,32]. Briefly, overlapping raw sequence reads were first merged to generate contigs that represented the V1–V3 regions of 16S rRNA genes that were PCR-amplified with the 27F and 519R primers. These V1–V3 contigs were then filtered for quality, aligned, and clustered into Operational Taxonomic Units (OTUs). OTUs were then curated by screening and removing artefacts. The closest valid relatives for the most abundant OTUs were identified by searches with blastn against the ‘refseq_rna’ database [32]. For analysis of alpha diversity, curated datasets were first rarefied to 15,000 sequences with custom Perl scripts. Using the MOTHUR (v.1.44.1) open-source software package [27], the alpha diversity indices ‘Observed OTUs’, ‘Chao’, ‘Ace’, ‘Shannon’ and ‘Simpson’ were determined using the ‘summary.single’ command.

### 2.6. Statistical Analyses

The research was conducted as a completely randomized design. Repeated data were analyzed using the MIXED procedure of SAS (SAS software version 9.4, SAS Institute, Cary, NC). Lambs were treated as a random independent variable; treatment day and their interaction were treated as fixed effects. Weights, IgG concentrations (total and relative), percentage IgG remaining, and ovalbumin antibody S:P ratio were the dependent variables. Lamb, treatment, and time were included in the class statement; time was included in the repeated statement; and dependent variables were included in the model statement. Least Square Means were separated using the PDIFF option of the LSMEANS statement when interactions *p* values were >0.05 but ≤0.10.

Statistical testing of bacterial composition data was performed in ‘R’ (Version 3.6.0). A *t*-test was used to compare alpha diversity indices (parametric), while a Wilcoxon rank-sum test was used to compare OTU abundance data (non-parametric).

For all data collected, *p* values ≤ 0.05 are considered significant. Tendencies are described when *p* values are >0.05 but ≤0.10.

## 3. Results

### 3.1. Colostrum IgG Concentrations

Concentrations of IgG in frozen ewe colostrum, frozen cattle colostrum, artificial colostrum, and fresh colostrum sources are shown in Table 1.

### 3.2. Lamb Performance

Lamb growth performance is shown in Table 2. There were no differences (*p* ≥ 0.40) between lamb weights or average daily gain between treatments throughout the experiment.

### 3.3. IgG Levels

#### 3.3.1. Validation of Species-Specific IgG Detection

Ovine IgG radial immunodiffusion plates showed cross-reactivity with bovine IgG plates. Therefore, it was not possible to provide an accurate representation of ovine IgG concentrations in the groups receiving colostrum containing bovine IgG (AC and CC). However, bovine IgG radial immunodiffusion plates did not show cross-reactivity with ovine IgG. The Ovine IgG ELISA did not provide consistent results and showed false-positive results in known IgG-negative samples. Additionally, Bovine IgG ELISAs cross-reacted with Ovine IgG. Therefore, the ELISA tests used in this study were not valid and the results were not included.

#### 3.3.2. Serum IgG Levels

**Figure 1:**
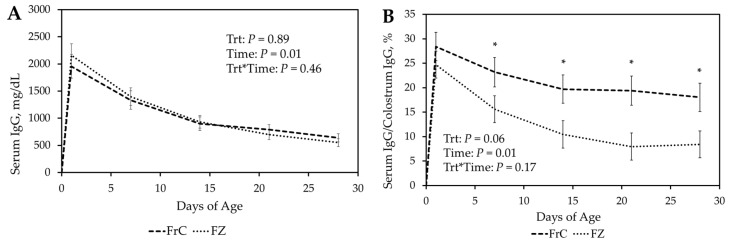

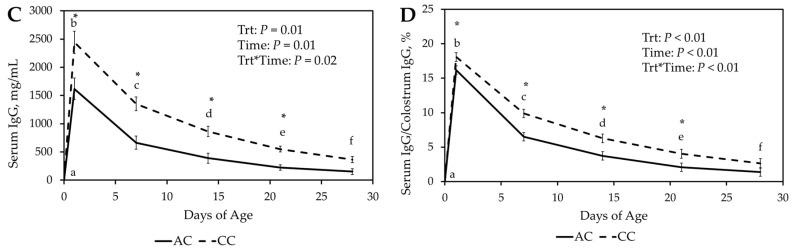
(**A**). Total serum concentration of ovine immunoglobulin G (IgG) in lambs receiving fresh ewe colostrum (FrC) compared to lambs receiving frozen ewe colostrum (FZ). (**B**). Relative ovine IgG (serum IgG concentration/colostrum IgG concentration) of lambs receiving FrC and FZ. * *p* ≤ 0.05 between treatments (unprotected F test by day). (**C**). Total serum bovine IgG concentrations of cattle colostrum (CC) and artificial colostrum (AC) groups. (**D**). Relative bovine IgG concentrations (Serum IgG concentration/colostrum IgG concentration) of AC and CC. * *p* ≤ 0.05 between treatments. ^a–f^ *p* ≤ 0.05 between days. Figures describe the first 28 days of age.

There was no significant (*p* ≥ 0.18) treatment-by-time interaction or main effect treatment in IgG serum concentrations between FZ and FrC (Figure 1A). Similarly, both FrC and FZ showed similar serum IgG concentrations throughout 28 D (*p* ≥ 0.40). Total serum IgG decreased over time in both treatment groups (*p* = 0.01; Figure 1A).

Relative ovine IgG concentrations (serum IgG concentration/colostrum IgG concentration) for FrC and FZ were calculated. There was no significant (*p* ≥ 0.18) treatment by time interaction in relative IgG concentrations (Figure 1B). However, there was a tendency (*p* = 0.06) for a main effect of treatment with both treatments being similar (*p* = 0.36) 24 h after birth, but FrC tending (*p* = 0.07) to have greater relative IgG at 7 D and being greater (*p* ≤ 0.03) at 14 D, 21 D, and 28 D (Figure 1B).

There was a significant (*p* = 0.02) treatment-by-time interaction for bovine IgG concentrations with CC lambs having greater IgG (*p* ≤ 0.01) at 24 h, 7 D, 14 D, and 21 D (Figure 1C). Serum bovine IgG concentrations tended (*p* = 0.10) to be greater in the CC lambs at 28 D (Figure 1C).

Relative bovine IgG concentrations (serum IgG concentration/colostrum IgG concentration) of CC and AC were calculated. There was a significant treatment-by-time interaction (*p* ≤ 0.01) with CC lambs having greater relative IgG concentrations (*p* ≤ 0.03) at 24 h, 7 D, 14 D, and 21 D (Figure 1D). Relative concentrations were similar between AC and CC groups at 28 D (*p* = 0.15; Figure 1D).

**Figure 2:**
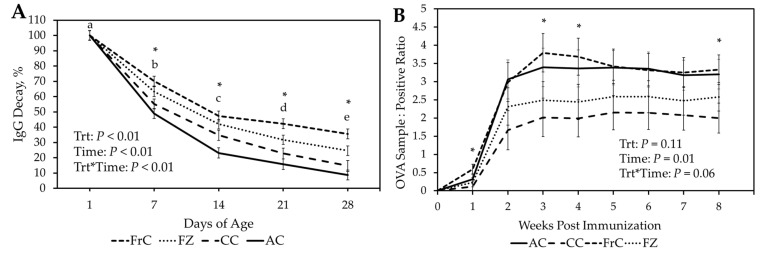
(**A**). Percent decay of serum Immunoglobulin G (IgG) concentration (Serum IgG by day/serum IgG concentration at day 1 of age) for Fresh ewe colostrum (FrC), frozen ewe colostrum (FZ), cattle colostrum (CC, and artificial colostrum (AC) treatment groups for the first 28 days of age. (**B**). Ovalbu-min antibody concentration in serum samples for AC, CC, FrC and FZ throughout 8 weeks post im-munization. * *p* ≤ 0.05 between treatments. ^a–e^ *p* ≤ 0.05 between days.

The percentage decay of serum IgG (Serum IgG by day/serum IgG concentration at day 1 of age) was calculated (Figure 2A, Table 3). There was a significant treatment-by-time interaction (*p* < 0.01) with FrC lambs having a greater percentage of IgG remaining than CC and AC throughout the first 28 D. The FrC group had also greater remaining IgG levels (*p* < 0.02) than FZ at 21 D and 28 D. The FZ group had greater (*p* < 0.01) remaining IgG levels than the AC group throughout the first 28 D. The FZ and CC groups were similar (*p* > 0.07) remaining IgG levels through the first 21 D; however, FC had greater remaining IgG levels (*p* = 0.03) at 28 D. The CC group had similar remaining IgG levels (*p* > 0.15) to the AC group at 7 D and 21 D, however CC had greater (*p* = 0.02) remaining IgG levels at 14 D.

### 3.4. Ovalbumin Challenge

All groups were confirmed seronegative to OVA prior to immunization. There was a tendency (*p* = 0.06) for treatment by time effect, with FrC lambs having greater antibody concentration at 1 (0.60 vs. 0.11 ± 0.13 S:P), 3 (3.79 vs. 2.02 ± 0.53 S:P), 4 (3.69 vs. 1.99 ± 0.51 S:P) and 8 (3.33 vs. 2.00 ± 0.41 S:P) weeks post immunization compared to CC (Figure 2B). Moreover, antibody concentration tended (*p* = 0.07) to be greater at 2, 5, 6, and 7 weeks post-immunization in FrC vs. CC lambs. Lambs fed AC had greater (*p* = 0.04) concentrations of antibodies than lambs receiving CC at 8 (3.21 vs. 2.00 ± 0.41 S:P) weeks post immunization. Lambs fed AC also tended (*p* = 0.07) to have greater concentrations of antibodies than lambs fed CC at 2, 3, 4, 5, 6, and 7 weeks post-immunization (Figure 2B).

### 3.5. Fecal Microbiome

To determine if there was a potential effect of freezing colostrum on the future development of gut bacterial communities, fecal samples were used as a proxy to compare bacterial composition between lambs that were fed fresh ewe colostrum and lambs that were fed frozen ewe colostrum. Fecal samples collected at day 28 from nine lambs from each treatment were used for this analysis. A combined total of 4247 bacterial OTUs were identified from a dataset consisting of 43,418–80,903 sequence reads per sample that targeted the V1-V3 region of the 16S rRNA gene. No significant differences in alpha diversity indices (Supplemental Table S1) or abundance of most abundant OTUs (Supplemental Table S2) were found between the two treatments.

## 4. Discussion

Results from this study demonstrated that fresh ewe colostrum provided the greatest relative IgG concentration and the slowest IgG decay. Lambs receiving fresh colostrum had a faster and higher antibody response to ovalbumin than lambs receiving cattle colostrum. The ovalbumin antibody response to artificial colostrum was similar to fresh colostrum, while lambs receiving frozen ewe colostrum had an intermediate ovalbumin antibody response.

Colostral IgG concentrations varied across colostral sources. To account for these differences, relative serum IgG was calculated by dividing the serum IgG concentration by the corresponding colostrum IgG concentration. To standardize colostrum volume across all lambs, individual lamb dose was 65 mL/kg, an amount similar to the approximate blood volume per unit of body weight in neonatal lambs [33]. This approach ensured consistency of the colostrum-to-blood volume ratio, regardless of birthweight. Additionally, serum IgG percent decay of each colostrum source was analyzed to quantify IgG remaining in the lamb relative to what they received.

Lamb serum IgG concentrations were greatest at 24 h in FrC vs. FZ, and in CC vs. AC. This, coupled with the increased rate of percent decay in bovine (CC and AC) vs. ovine (FrC and FZ), suggests that passive absorption occurs more efficiently with ovine IgG compared to bovine IgG. The increase in efficiency of passive transfer may be due to ovine IgG-specific uptake pathways in the enterocytes of newborn lambs [34]. One known mechanism involves the neonatal Fc receptor (FcRn), which specifically binds the Fc region of maternal IgG in the intestinal lumen and facilitates transcytosis across the enterocyte [34]. It is unclear whether bovine IgG molecules can bind and be transported by the ovine FcRn, or if they are transported mainly by nonspecific pathways like paracellular permeability or micropinocytosis [35,36]. The greater percentage of IgG absorbed in the CC group over the AC group could be a result of the greater concentration of IgG mg/dL in CC compared to AC, creating a stronger concentration gradient that could have increased passive transfer.

At 24 h, relative serum IgG concentrations remained greatest in the FrC group, followed by FZ, CC, and AC, respectively. Despite IgG in FZ being absorbed at the same rate as FrC, relative FZ IgG levels decreased at a faster rate than FrC. One possible explanation is that freezing colostrum may induce structural or functional damage that results in a reduced half-life of IgG in lambs. While the effects of freezing ewe colostrum on IgG concentration have not been evaluated, studies in sows and humans suggest that colostrum can be frozen for up to three months without significantly impacting IgG concentrations [37,38]. However, there is little information on how freezing affects the half-life of IgG once passively absorbed by the neonate. The half-life of IgG in lambs is expected to be around seven days [39]. Bovine IgG appears to have a similar half-life in lambs, as the AC and CC treatment groups both experienced a reduction of approximately half each week. The FrC and FZ treatment groups showed more sustained concentrations of IgG, potentially because ovine IgG had a longer half-life than bovine IgG in the neonates. Host production of IgG is another potential contributor to why the FrC group had more sustained relative IgG concentrations.

Colostrum contains multiple bioactive components including maternal leukocytes, cytokines, and hormones, all of which may impact immune development [11,12,40]. Studies in human colostrum indicate that cytokines are present after a freeze–thaw cycle [40]. However, cytokines usually have short half-lives [41], which suggests that a freeze–thaw process could significantly decrease their concentration in the colostrum. In calves, colostrum-derived pro-inflammatory cytokines promote neonate immune development by recruiting lymphocytes to the mucosal barrier [12]. Without the presence of these cytokines, FZ, CC, and AC groups could have experienced delayed immune development in this study. Furthermore, maternal leukocytes in the colostrum increase antigen-presenting cells, which promote the development of the adaptive immune response [11]. While maternal leukocytes can survive the freezing process with just a 10% death loss, their ability to perform the same cellular function post-thaw is unclear [38]. A reduction in maternal leukocyte function could decrease the efficiency of antigen-presenting cells and result in delayed immune development. Unfortunately, the ovine IgG RID plates cross-reacting with bovine IgG prevented us from measuring host production of IgG in the AC and CC groups. More precise IgG detection techniques that allow for separate detection of bovine IgG and ovine IgG would enable us to have a more complete understanding of how bovine colostrum sources impact host production of IgG early in neonatal life.

The FrC group showed a more rapid and robust antibody response to the ovalbumin challenge compared to those receiving CC. As it was observed with the relative IgG data, it appears that the lambs receiving FrC were capable of synthesizing IgG molecules earlier, which coincides with the increased ovalbumin-specific antibodies seen in this study. This is likely due to the factors outlined previously, i.e., cytokines, hormones, and maternal leukocytes present in the fresh ewe colostrum. These immune system stimulatory factors could have fostered earlier immune development, which would result in the more rapid and robust response observed. Another possibility is that the bovine IgG received by the CC and AC groups bound to pathogens, and the fragment crystallizable region of the bovine IgG molecule was unable to interact with host immune cells. This would result in suppressed development because host immune cells would have delayed exposure to pathogens, and consequently, the antibody-activated presentation pathways would not be stimulated. Some ability for cross-species interaction between the Fc region of IgG and the host Fc receptor has been demonstrated in human and mouse models [42]. More research is needed to understand the interaction between the Fc regions of bovine IgG and the ovine Fc IgG receptor. Interestingly, the AC treatment group showed a similar response to the ovalbumin compared to the FrC group, even in the absence of the bioactive factors outlined above. This indicates that there are likely colostrum-independent pathways for adaptive immune development. One potential explanation is the decreased inhibition of maternal antibodies that may limit the neonate’s immune system from being exposed to pathogens. The AC colostrum group had the lowest concentration of antibodies present for the first 28 days of life. This low concentration of “maternal” antibodies would have coated fewer pathogens, which would have increased the lamb’s exposure to uncoated pathogens earlier in life, resulting in earlier development of the adaptive immune system. Studies in calves reported no differences in response to ovalbumin between colostrum-fed and colostrum-deprived calves [43]. These findings, alongside the response from the AC treatment group, support the hypothesis that when lower levels of maternal immunoglobulins are present, neonatal ruminants can develop their adaptive immune system via colostrum-independent pathways.

There was no impact of colostrum on lamb weights at weaning or 90 days of age. Previous studies have reported that serum IgG concentration is positively correlated with pre-weaning average daily gain [44]. Data obtained from this project did not support this hypothesis; however, it is worth considering that most of the lambs in this trial remained healthy and were housed in clean, dry pens, with feeders cleaned regularly. Different lamb production systems have different housing and feeders cleansing and disinfecting routines; thus, these differences may affect the control of environmental pathogens that can cause subclinical diseases that can affect lamb performance [45].

## 5. Conclusions

Findings from this project demonstrate that in Polypay sheep fresh ewe colostrum provides the most effective passive immune protection and stimulates the development of the humoral immune system better than other colostrum sources. Ovine IgG appears to be absorbed more efficiently and sustained in the serum of the neonate for longer than bovine IgG, potentially due to species-specific uptake pathways and enhanced immunostimulatory activity from other bioactive components. Frozen ewe colostrum was the best out of the frozen and artificial substitutes at providing passive immune protection. The influence of maternal leukocytes, cytokines, and hormones present in the colostrum likely resulted in earlier and stronger immune responses observed in the FrC group. These results showcase the importance of host-derived colostrum sources and highlight some of the limitations of utilizing bovine or bovine processed colostrum substitutes.

The limitations of this study include the limited sample size (*n* = 43 total, with a microbiome subset of *n* = 18), which may affect the generalizability of the findings. Furthermore, interpretation of tendencies (*p* > 0.05 but ≤0.10) should be considered exploratory and requires cautious interpretation. Supplementary research should focus on the effects of similar colostrum sources on both cellular and humoral adaptive immunity using a larger sample size. Additionally, future research in detecting differences in host vs. colostrum-derived IgG, as well as characterization of other immune components, will provide better insight into understanding lamb immune development.

## Figures and Tables

**Table 1:** Immunoglobulin G concentrations in pooled frozen ewe colostrum (FZ), pooled frozen cattle colostrum (CC), artificial colostrum (AC) and fresh colostrum (FrC) sources.

Treatment Group	* IgG (mg/mL) ± SEM
Frozen Ewe Colostrum	87.47 ± 1.52
Frozen Cattle Colostrum	139.37 ± 4.22
Artificial Colostrum	89.99 ± 3.36
Fresh Ewe Colostrum ^1^	
Dam 1	78.53 ± 0.12
Dam 2	98.86 ± 3.15
Dam 3	83.15 ± 0.00
Dam 4	54.84 ± 2.41
Dam 5	59.27 ± 4.09

* All samples were analyzed in duplicate, average IgG values ± SEM is presented. ^1^ Fresh ewe colostrum was collected from 5 ewes and is shown as individual values to represent the values of IgG that FrC lambs received.

**Table 2:** Growth performance of lambs receiving fresh colostrum (FrC), frozen colostrum (FZ), cattle colostrum (CC), and artificial colostrum (AC).

Treatment Groups						
	FrC (*n* = 10)	FZ (*n* = 11)	CC (*n* = 11)	AC (*n* = 11)	SEM ^1^	*p*-Value
Variable						
BW ^2^, kg	5.13	4.64	4.62	4.79	0.33	0.40
WWT ^3^, kg	16.71	15.66	16.10	15.59	0.85	0.54
PWWT ^4^, kg	35.09	33.77	32.42	32.91	1.91	0.51
PrWADG ^5^, kg/d	0.39	0.37	0.38	0.36	0.05	0.55
PoWADG ^6^, kg/d	0.28	0.25	0.27	0.26	0.07	0.77

^1^ Standard Error of the Mean. ^2^ Birth Weight. ^3^ Weaning Weight. ^4^ Post Weaning Weight measured at 90 days of age. ^5^ Pre-weaning average daily gain. ^6^ Post-weaning average daily gain.

**Table 3:** Percent decay of serum Immunoglobulin G (IgG) concentration (Serum IgG by day/serum IgG concentration at day 1 of age) for Fresh ewe colostrum (FrC), frozen ewe colostrum (FZ), cattle colostrum (CC, and artificial colostrum (AC) treatment groups for the first 28 days of age.

Treatment Groups					*p*-Value			
WOA ^1^	FrC (*n* = 10)	FZ (*n* = 11)	CC (*n* = 11)	AC (*n* = 11)	SEM ^2^	Trt	Time	Trt * Time
1	70.14 ^a^	63.27 ^ab^	55.24 ^bc^	48.92 ^c^	3.21	<0.01	<0.01	<0.01
2	47.35 ^a^	41.98 ^ab^	34.78 ^b^	23.14 ^c^	3.36			
3	42.31 ^a^	31.79 ^bc^	22.83 ^cd^	15.81 ^d^	3.53			
4	35.64 ^a^	24.62 ^b^	14.76 ^c^	8.71 ^c^	3.53			

^1^ Weeks of Age. ^2^ Standard Error of the Mean. ^a–d^ *p* ≤ 0.05 between treatments within a week.

## Data Availability

The original contributions presented in this study are included in the article/Supplementary Material. Further inquiries can be directed to the corresponding author.

## Supplementary Materials

The following supporting information can be downloaded at: https://www.mdpi.com/article/10.3390/vetsci12111075/s1, Table S1: Alpha-diversity indices of fecal bacterial communities (day 28) of lambs that received fresh (*n* = 9) or frozen (*n* = 9) ewe colostrum at birth. Values are presented as mean of abundance and standard error of the mean, respectively, Table S2: Most abundant OTUs from fecal bacterial communities (day 28) of lambs that received fresh (*n* = 9) or frozen (*n* = 9) ewe colostrum at birth. Values are presented as mean of abundance and standard error of the mean, respectively. Most abundant OTUs were defined as having a mean of at least 1% in at least one of the two treatment groups.

## Abbreviations

The following abbreviations are used in this manuscript:

IgG	Immunoglobulin G
FrC	Fresh ewe colostrum
FZ	Frozen ewe colostrum
CC	Frozen bovine colostrum
AC	Artificial bovine colostrum
D	Days of age
ELISA	Enzyme-Linked Immunosorbent Assay
RID	Radial Immunodiffusion
OVA	Ovalbumin
BAD	Blocking antibody diluent
PBS	Phosphate-buffered saline
PBST	Phosphate-buffered saline + 0.01% Tween 20
OD	Optical densities
OTU	Operational Taxonomic Unit
FcRn	Neonatal Fc receptor
