# Supplementary material for: Evaluation of the Effects of Colostrum Substitutes on IgG Levels and Humoral Immune Development in Polypay Lambs
Source: Vet Sci. 2025 Nov 10;12(11):1075. doi: 10.3390/vetsci12111075 (PMC12656888; doi:10.3390/vetsci12111075)
Supplement: Supplementary file 1 [file vetsci-12-01075-s001.zip › vetsci-3863122-supplementary.pdf]

Supplemental Table S1. Alpha-diversity indices of fecal bacterial communities (day 28) of lambs that received fresh (n=9) or frozen (n=9) ewe colostrum at birth. Values are presented as mean of abundance and standard error of the mean, respectively.

Diversity indices	Fresh colostrum	Frozen colostrum	P value
Observed OTUs	476.8 ± 27.2	474.9 ± 19.8	0.95
Chao	1042.9 ± 67.8	1016.4 ± 55.2	0.76
Ace	1697.3 ± 128.5	1755.3 ± 165.0	0.78
Shannon	3.30 ± 0.16	3.26 ± 0.12	0.87
Simpson	0.12 ± 0.02	0.11 ± 0.02	0.85
Coverage	0.98 ± <0.01	0.98 ± <0.01	NA

Supplementary methods:

Sequences were merged from overlapping paired end reads using the 'make.contigs' command from the MOTHUR (v.1.44.1) open-source software package [27]; these contigs corresponded to V1–V3 amplicons generated from the 16S rRNA bacterial gene. Contig sequences for the V1–V3 region were first screened to meet the following criteria: presence of both intact 27F and 519R primer sequences, a minimal average Phred quality score of Q33, and length between 400 and 580 nt. After quality screening, V1–V3 sequences were aligned, then clustered into Operational Taxonomic Units (OTUs) using a sequence dissimilarity cutoff of 4%. This threshold is more suitable for the V1–V3 region than the 3% cutoff that is typically used indiscriminately for clustering of 16S rRNA sequence data, regardless of the variable regions targeted for analysis; for further details, please refer to Kim et al. [28] and Johnson et al. [29]. Three different approaches were then used to screen for sequence artifacts. Operational Taxonomic Units were screened for chimeric sequences using the 'chimera.slayer' [30] and 'chimera.uchime' [31] commands in MOTHUR (v.1.44.1) [27]. The 5′ and 3′ ends of OTUs were also evaluated using a database alignment search-based approach; when compared to their closest match of equal or longer sequence length from the NCBI 'nt' database, as determined by blastn [32], OTUs with more than five nucleotides missing from the 5′ or 3′ end of their respective alignments were designated as artifacts. Finally, OTUs with only one or two assigned reads were subjected to an additional screen, where only sequences with a perfect or near-perfect match (maximum 1% of dissimilar nucleotides) to a sequence in the NCBI 'nt' database were kept for analysis. All OTUs and their assigned reads that were flagged as artifacts during these screens were subsequently removed from further analyses. The closest valid relatives for the most abundant OTUs were identified by searches with blastn against the 'refseq_rna' database [32].

Supplementary Table S2. Most abundant OTUs from fecal bacterial communities (day 28) of lambs that received fresh (n=9) or frozen (n=9) ewe colostrum at birth. Values are presented as mean of abundance and standard error of the mean, respectively. Most abundant OTUs were defined as having a mean of at least 1% in at least one of the two treatment groups.

OTU	Fresh colostrum	Frozen colostrum	Closest valid taxon (id%)	P value
Oa-00101	17.16 ± 5.08	16.15 ± 5.16	*Phocaeicola vulgatus* (99.05%)	0.93
Oa-00102	4.15 ± 1.30	7.06 ± 2.51	*Blautia hansenii* (99.03%)	0.80
Oa-00103	6.99 ± 2.85	2.67 ± 1.65	*Limosilactobacillus balticus* (99.46%)	0.30
Oa-00104	2.70 ± 0.93	2.38 ± 1.80	*Bacteroides stercoris* (99.61%)	0.06
Oa-00105	1.74 ± 0.56	3.73 ± 2.13	*Allofournierella massiliensis* (98.84%)	0.93
Oa-00106	3.69 ± 2.53	2.14 ± 1.40	*Escherichia fergusonii* (98.67%)	0.80
Oa-00107	4.39 ± 4.30	0.16 ± 0.07	*Helicobacter pullorum* (98.76%)	0.67
Oa-00108	2.32 ± 0.78	3.41 ± 1.37	*Mediterraneibacter gnavus* (98.42%)	0.67
Oa-00109	3.19 ± 1.62	1.93 ± 0.75	*Butyricicoccus pullicaecorum* (95.95%)	1.00
Oa-00110	2.52 ± 1.41	2.26 ± 0.91	*Streptococcus pasteurianus* (100.00%)	0.93
Oa-00111	2.14 ± 0.90	1.26 ± 0.83	*Faecalibacterium hattorii* (98.01%)	0.39
Oa-00112	0.54 ± 0.23	4.26 ± 4.07	*Gemmiger gallinarum* (94.82%)	0.44
Oa-00113	1.23 ± 0.42	2.34 ± 1.24	*Coprococcus phoceensis* (98.45%)	0.86
Oa-00114	0.93 ± 0.21	2.94 ± 1.00	*Thomasclavelia spiroformis* (99.42%)	0.05
Oa-00115	1.94 ± 1.10	0.96 ± 0.21	*Bacteroides fragilis* (99.43%)	0.86
Oa-00116	3.38 ± 1.56	0.82 ± 0.78	*Bifidobacterium longum* (99.19%)	0.30
Oa-00117	0.81 ± 0.70	2.90 ± 1.80	*Faecalicoccus pleomorphus* (97.95%)	0.73
Oa-00118	1.52 ± 0.59	0.68 ± 0.31	*Sutterella massiliensis* (98.85%)	0.11
Oa-00119	0.48 ± 0.15	1.68 ± 1.15	*Pseudoruminococcus massiliensis* (83.43%)	0.39
Oa-00120	2.78 ± 2.76	0.02 ± <0.01	*Clostridium innocuum* (92.41%)	0.30
Oa-00121	0.82 ± 0.29	1.23 ± 0.50	*Faecalimonas umbilicate* (93.98%)	0.86
Oa-00122	1.20 ± 0.21	0.75 ± 0.20	*Phocaeicola vulgatus* (96.01%)	0.08
Oa-00123	0.93 ± 0.30	1.04 ± 0.43	*Blautia producta* (94.95%)	0.93
Oa-00124	1.42 ± 0.71	0.35 ± 0.17	*Faecalibacterium langellae* (99.20%)	0.16
Oa-00125	0.65 ± 0.22	1.20 ± 0.32	*Coprococcus phoceensis* (95.16%)	0.26
Oa-00126	1.38 ± 0.57	0.53 ± 0.33	*Limosilactobacillus balticus* (99.11%)	0.39
Oa-00128	0.02 ± 0.19	1.58 ± 0.32	*Gluceribacter canis* (97.79%)	0.49
Oa-00129	0.77 ± 0.00	1.22 ± 1.49	*Blautia hansenii* (97.28%)	0.55
Oa-00130	0.54 ± 0.23	1.59 ± 0.34	*Blautia caecimuris* (99.55%)	0.67
Oa-00132	0.75 ± 0.31	1.26 ± 0.85	*Blautia glucerasea* (96.11%)	0.93
Oa-00138	0.13 ± 0.20	1.21 ± 0.45	*Enterocloster bolteae* (93.98%)	0.44
Oa-00139	1.01 ± 0.39	0.15 ± 0.62	*Lactobacillus johnsonii* (99.45%)	0.14
